# Supplementary material for: The Translocator Protein (TSPO) Genetic Polymorphism A147T Is Associated with Worse Survival in Male Glioblastoma Patients
Source: Cancers (Basel). 2021 Sep 8;13(18):4525. doi: 10.3390/cancers13184525 (PMC8471762; doi:10.3390/cancers13184525)
Supplement: Supplementary file 1 [file cancers-13-04525-s001.zip › Supplementary Material/Supplementary Figure-1 08-25-2021.pptx]

## Slide 1
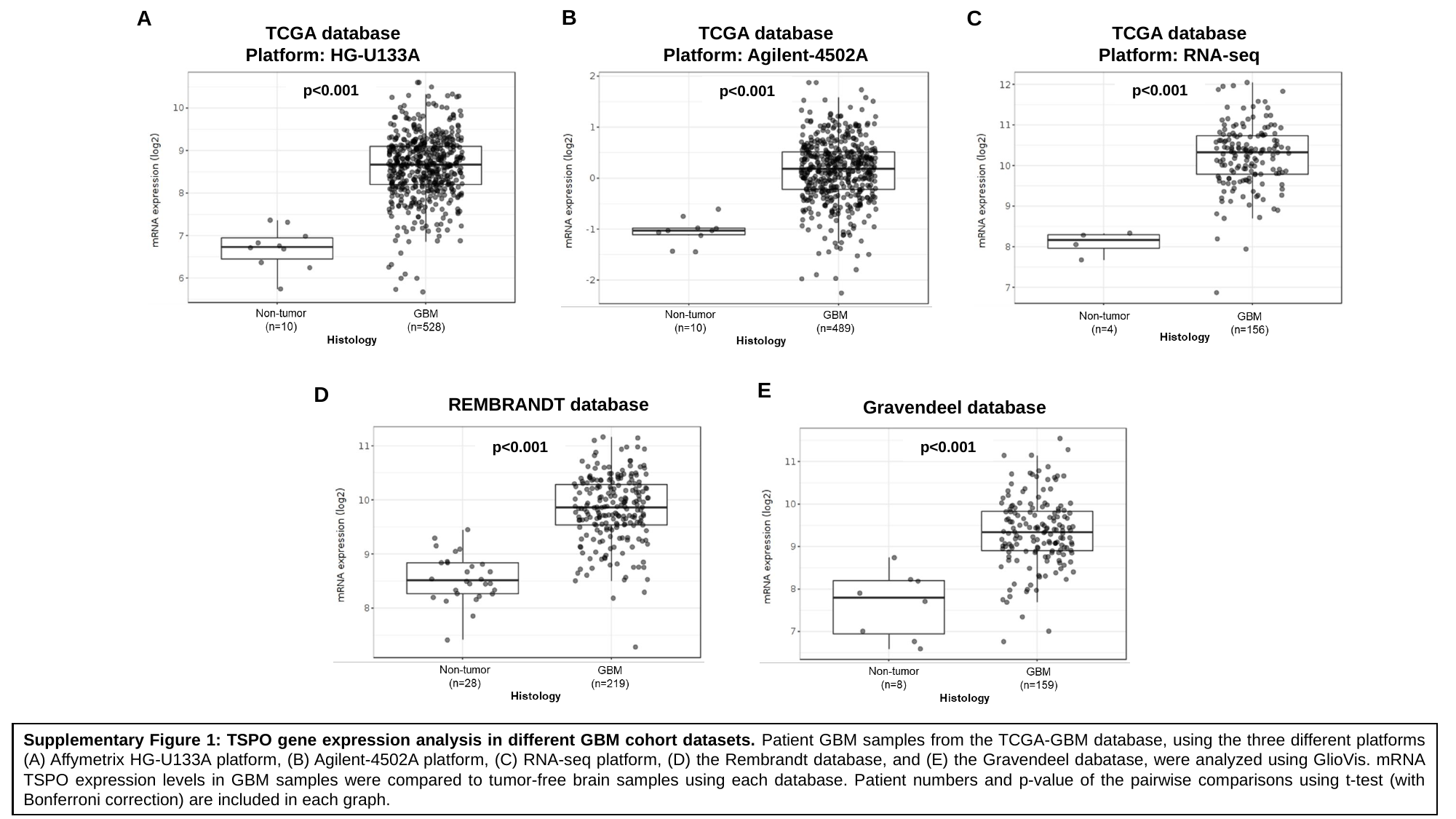

A
C
B
TCGA database
Platform: RNA-seq
TCGA database
Platform: Agilent-4502A
TCGA database
Platform: HG-U133A
E
D
REMBRANDT database
Gravendeel database
p<0.001
p<0.001
p<0.001
p<0.001
p<0.001
Supplementary Figure 1: TSPO gene expression analysis in different GBM cohort datasets. Patient GBM samples from the TCGA-GBM database, using the three different platforms (A) Affymetrix HG-U133A platform, (B) Agilent-4502A platform, (C) RNA-seq platform, (D) the Rembrandt database, and (E) the Gravendeel dabatase, were analyzed using GlioVis. mRNA TSPO expression levels in GBM samples were compared to tumor-free brain samples using each database. Patient numbers and p-value of the pairwise comparisons using t-test (with Bonferroni correction) are included in each graph.
