# Supplementary material for: The Translocator Protein (TSPO) Genetic Polymorphism A147T Is Associated with Worse Survival in Male Glioblastoma Patients
Source: Cancers (Basel). 2021 Sep 8;13(18):4525. doi: 10.3390/cancers13184525 (PMC8471762; doi:10.3390/cancers13184525)
Supplement: Supplementary file 1 [file cancers-13-04525-s001.zip › Supplementary Material/Supplementary Figure-2 08-25-2021.pptx]

## Slide 1
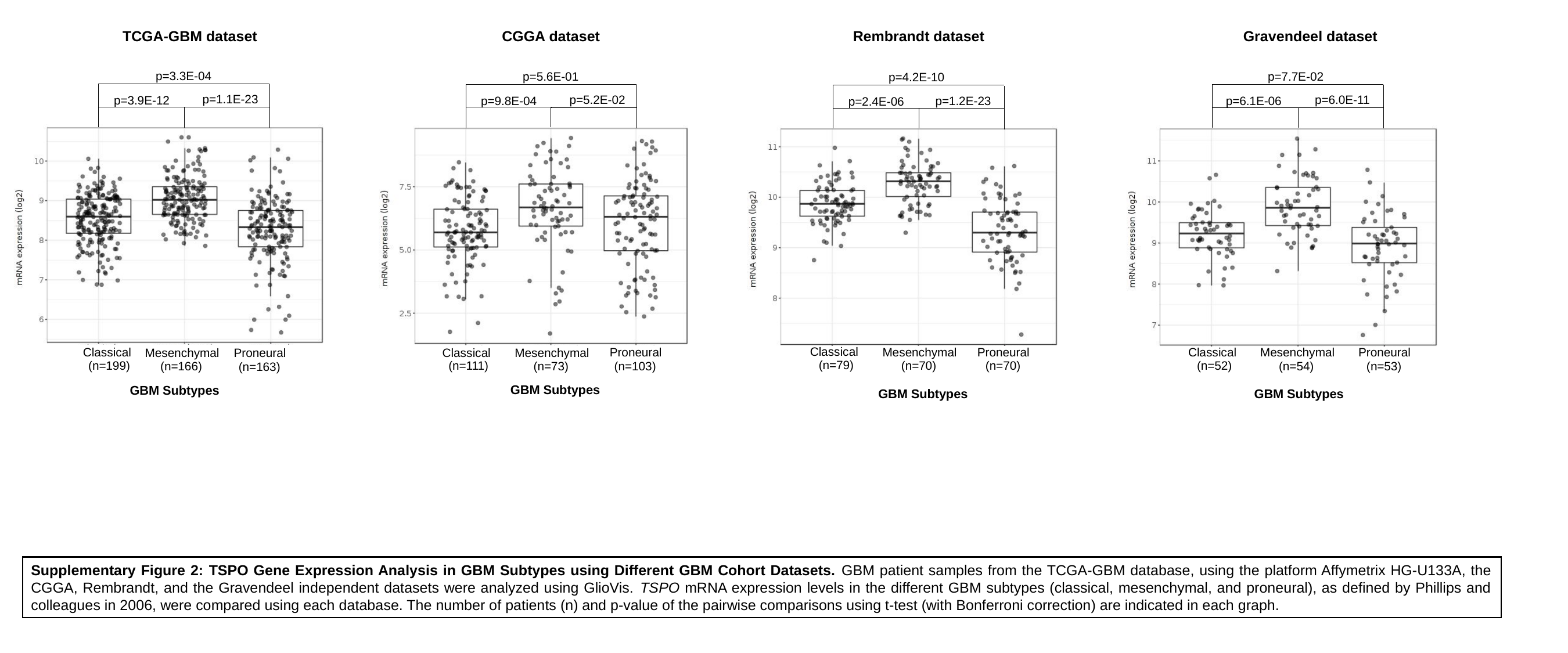

Gravendeel dataset
CGGA dataset
Rembrandt dataset
TCGA-GBM dataset
p=3.3E-04
p=5.6E-01
p=7.7E-02
p=4.2E-10
p=1.1E-23
p=5.2E-02
p=6.0E-11
p=3.9E-12
p=1.2E-23
p=9.8E-04
p=6.1E-06
p=2.4E-06
Classical
Proneural
Mesenchymal
(n=199)
(n=166)
(n=163)
GBM Subtypes
Proneural
Mesenchymal
Classical
(n=111)
(n=73)
(n=103)
GBM Subtypes
Classical
Proneural
Mesenchymal
(n=79)
(n=70)
(n=70)
GBM Subtypes
Proneural
Mesenchymal
Classical
(n=52)
(n=54)
(n=53)
GBM Subtypes
Supplementary Figure 2: TSPO Gene Expression Analysis in GBM Subtypes using Different GBM Cohort Datasets. GBM patient samples from the TCGA-GBM database, using the platform Affymetrix HG-U133A, the CGGA, Rembrandt, and the Gravendeel independent datasets were analyzed using GlioVis. TSPO mRNA expression levels in the different GBM subtypes (classical, mesenchymal, and proneural), as defined by Phillips and colleagues in 2006, were compared using each database. The number of patients (n) and p-value of the pairwise comparisons using t-test (with Bonferroni correction) are indicated in each graph.
