# Supplementary material for: The Translocator Protein (TSPO) Genetic Polymorphism A147T Is Associated with Worse Survival in Male Glioblastoma Patients
Source: Cancers (Basel). 2021 Sep 8;13(18):4525. doi: 10.3390/cancers13184525 (PMC8471762; doi:10.3390/cancers13184525)
Supplement: Supplementary file 1 [file cancers-13-04525-s001.zip › Supplementary Material/Supplementary Figure-3_08-25-2021.pptx]

## Slide 1
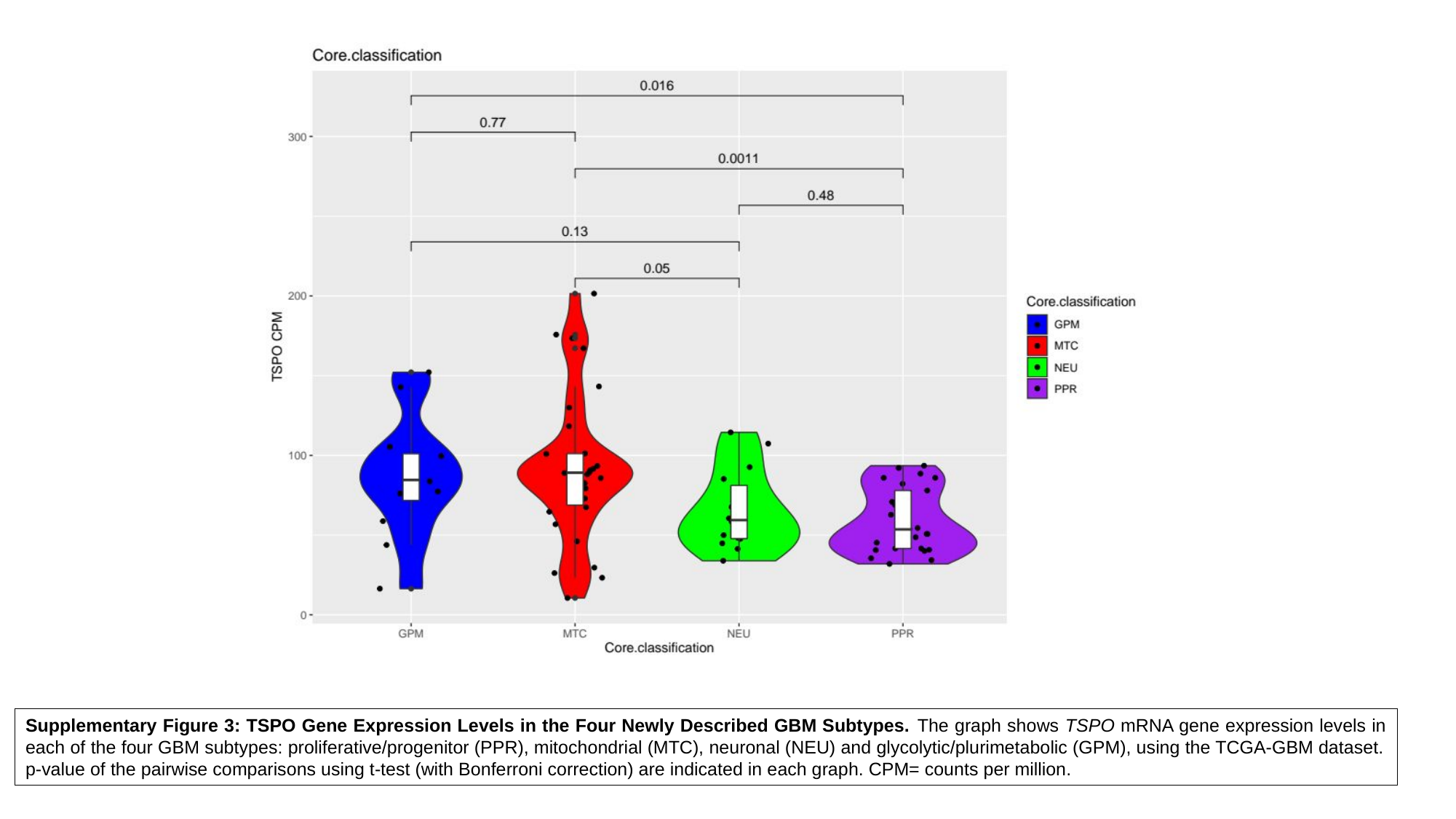

Supplementary Figure 3: TSPO Gene Expression Levels in the Four Newly Described GBM Subtypes. The graph shows TSPO mRNA gene expression levels in each of the four GBM subtypes: proliferative/progenitor (PPR), mitochondrial (MTC), neuronal (NEU) and glycolytic/plurimetabolic (GPM), using the TCGA-GBM dataset. p-value of the pairwise comparisons using t-test (with Bonferroni correction) are indicated in each graph. CPM= counts per million.
