# Supplementary material for: The Translocator Protein (TSPO) Genetic Polymorphism A147T Is Associated with Worse Survival in Male Glioblastoma Patients
Source: Cancers (Basel). 2021 Sep 8;13(18):4525. doi: 10.3390/cancers13184525 (PMC8471762; doi:10.3390/cancers13184525)
Supplement: Supplementary file 1 [file cancers-13-04525-s001.zip › Supplementary Material/Supplementary Figure-4_08-25-2021.pptx]

## Slide 1
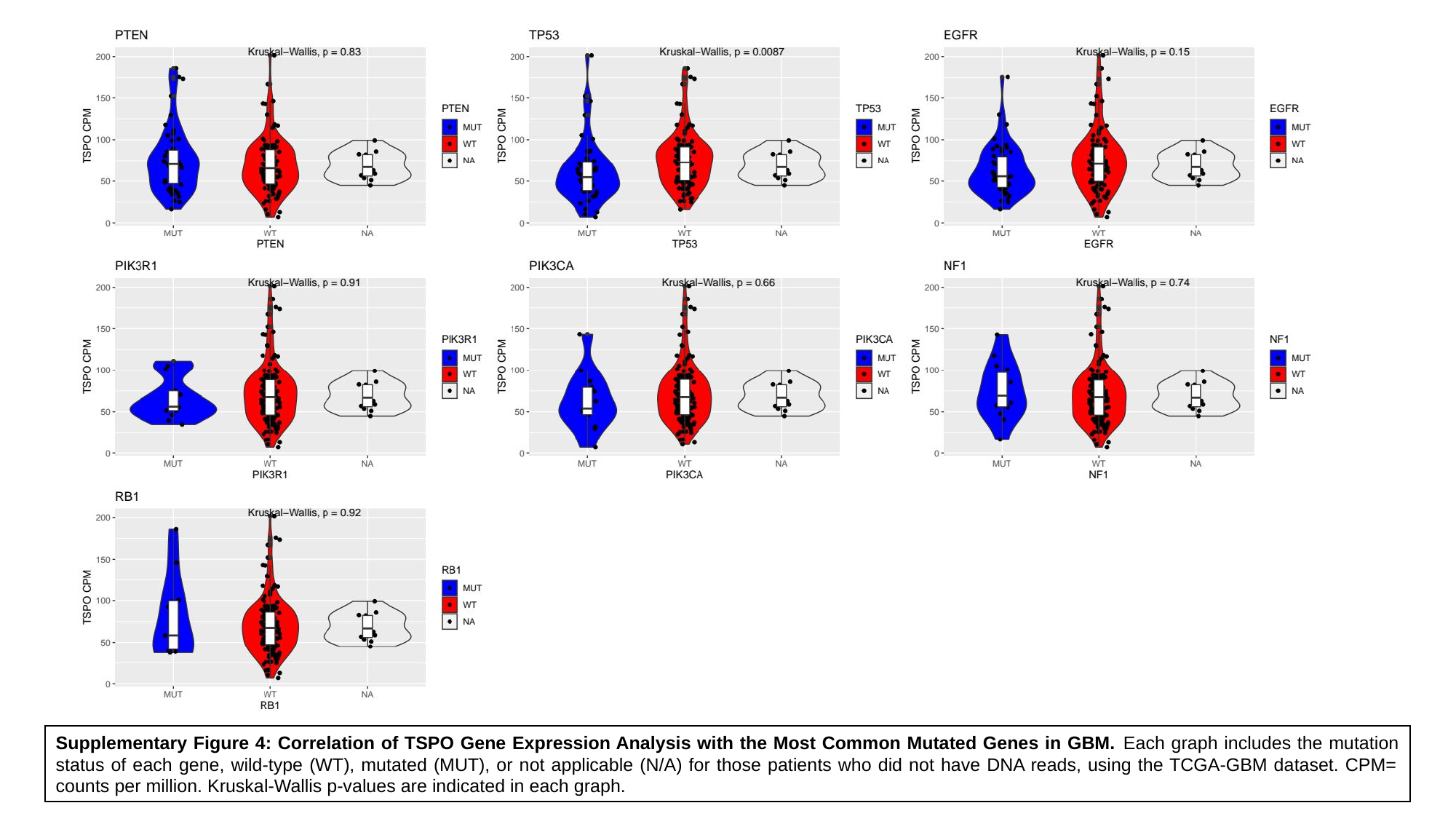

Supplementary Figure 4: Correlation of TSPO Gene Expression Analysis with the Most Common Mutated Genes in GBM. Each graph includes the mutation status of each gene, wild-type (WT), mutated (MUT), or not applicable (N/A) for those patients who did not have DNA reads, using the TCGA-GBM dataset. CPM= counts per million. Kruskal-Wallis p-values are indicated in each graph.
