# Supplementary material for: The Translocator Protein (TSPO) Genetic Polymorphism A147T Is Associated with Worse Survival in Male Glioblastoma Patients
Source: Cancers (Basel). 2021 Sep 8;13(18):4525. doi: 10.3390/cancers13184525 (PMC8471762; doi:10.3390/cancers13184525)
Supplement: Supplementary file 1 [file cancers-13-04525-s001.zip › Supplementary Material/Supplementary Figure-5_08-25-2021.pptx]

## Slide 1
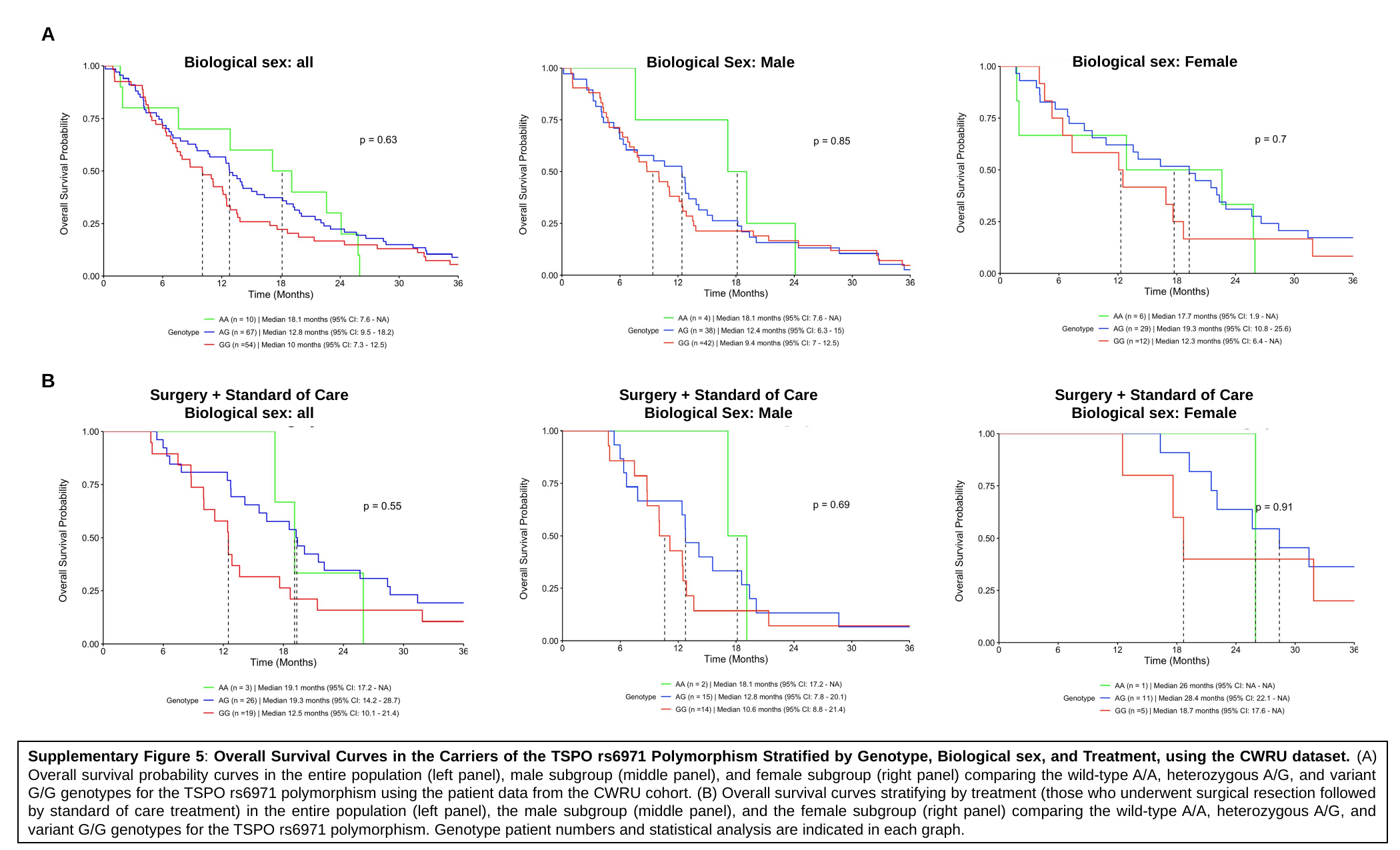

A
Biological sex: Female
Biological sex: all
Biological Sex: Male
B
Surgery + Standard of Care
Biological sex: all
Surgery + Standard of Care
Biological Sex: Male
Surgery + Standard of Care
Biological sex: Female
Supplementary Figure 5: Overall Survival Curves in the Carriers of the TSPO rs6971 Polymorphism Stratified by Genotype, Biological sex, and Treatment, using the CWRU dataset. (A) Overall survival probability curves in the entire population (left panel), male subgroup (middle panel), and female subgroup (right panel) comparing the wild-type A/A, heterozygous A/G, and variant G/G genotypes for the TSPO rs6971 polymorphism using the patient data from the CWRU cohort. (B) Overall survival curves stratifying by treatment (those who underwent surgical resection followed by standard of care treatment) in the entire population (left panel), the male subgroup (middle panel), and the female subgroup (right panel) comparing the wild-type A/A, heterozygous A/G, and variant G/G genotypes for the TSPO rs6971 polymorphism. Genotype patient numbers and statistical analysis are indicated in each graph.
