# Supplementary material for: The Translocator Protein (TSPO) Genetic Polymorphism A147T Is Associated with Worse Survival in Male Glioblastoma Patients
Source: Cancers (Basel). 2021 Sep 8;13(18):4525. doi: 10.3390/cancers13184525 (PMC8471762; doi:10.3390/cancers13184525)
Supplement: Supplementary file 1 [file cancers-13-04525-s001.zip › Supplementary Material/Supplementary Figure-6_08-25-2021.pptx]

## Slide 1
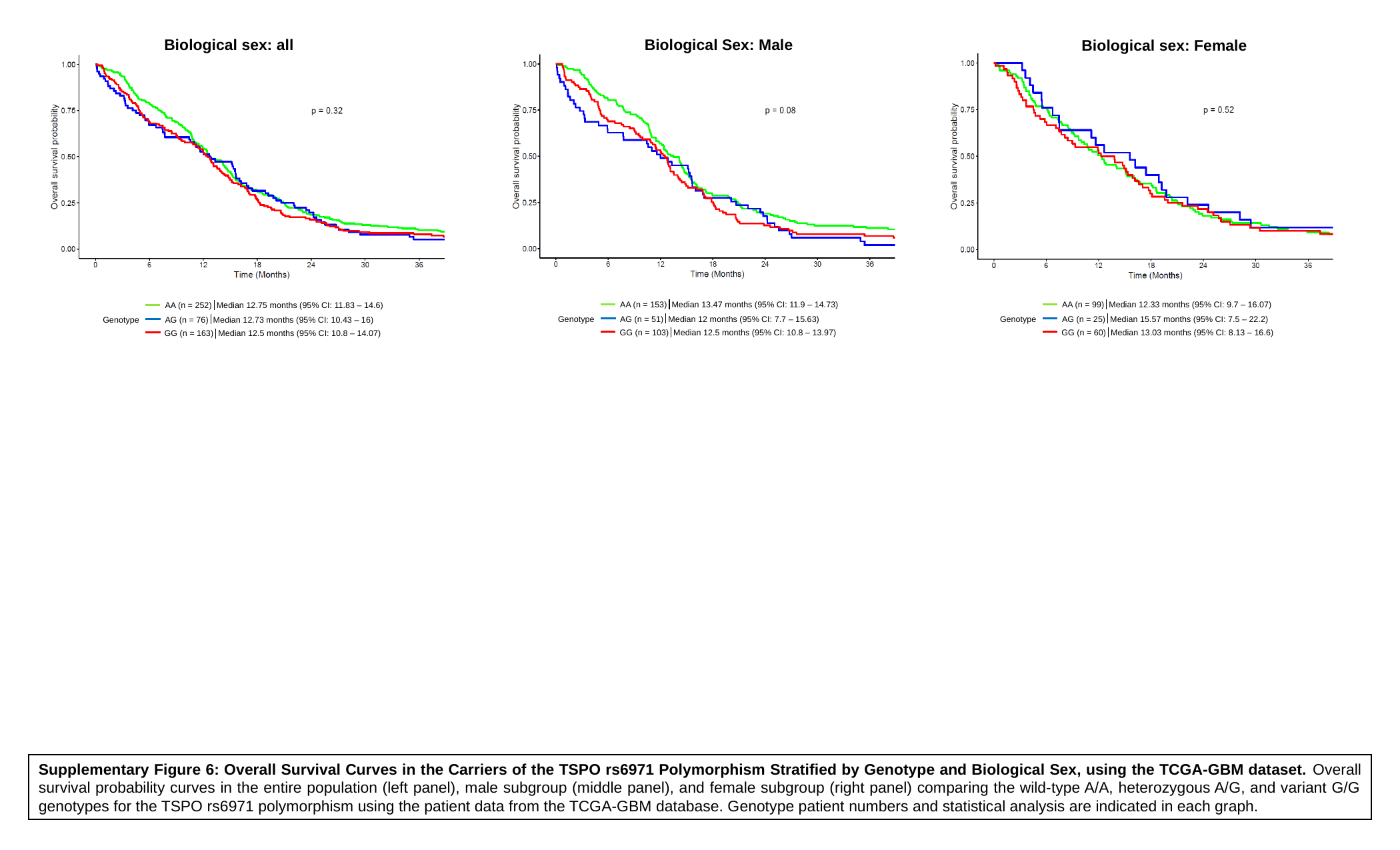

Biological sex: all
Biological Sex: Male
Biological sex: Female
AA (n = 153) Median 13.47 months (95% CI: 11.9 – 14.73)
AG (n = 51) Median 12 months (95% CI: 7.7 – 15.63)
Genotype
GG (n = 103) Median 12.5 months (95% CI: 10.8 – 13.97)
AA (n = 99) Median 12.33 months (95% CI: 9.7 – 16.07)
AG (n = 25) Median 15.57 months (95% CI: 7.5 – 22.2)
Genotype
GG (n = 60) Median 13.03 months (95% CI: 8.13 – 16.6)
AA (n = 252) Median 12.75 months (95% CI: 11.83 – 14.6)
AG (n = 76) Median 12.73 months (95% CI: 10.43 – 16)
Genotype
GG (n = 163) Median 12.5 months (95% CI: 10.8 – 14.07)
Supplementary Figure 6: Overall Survival Curves in the Carriers of the TSPO rs6971 Polymorphism Stratified by Genotype and Biological Sex, using the TCGA-GBM dataset. Overall survival probability curves in the entire population (left panel), male subgroup (middle panel), and female subgroup (right panel) comparing the wild-type A/A, heterozygous A/G, and variant G/G genotypes for the TSPO rs6971 polymorphism using the patient data from the TCGA-GBM database. Genotype patient numbers and statistical analysis are indicated in each graph.
